# Self-reported neurocognitive complaints in the Swiss HIV Cohort Study: a viral genome-wide association study

**DOI:** 10.1093/braincomms/fcae188

**Published:** 2024-05-31

**Authors:** Marius Zeeb, Chloé Pasin, Matthias Cavassini, Mélanie Bieler-Aeschlimann, Paul Frischknecht, Katharina Kusejko, Jacques Fellay, François Blanquart, Karin J Metzner, Kathrin Neumann, Lisa Jörimann, Jasmin Tschumi, Enos Bernasconi, Michael Huber, Helen Kovari, Karoline Leuzinger, Julia Notter, Matthieu Perreau, Andri Rauch, Alban Ramette, Marcel Stöckle, Sabine Yerly, Huldrych F Günthard, Roger D Kouyos, the Swiss HIV Cohort Study, Irene Abela, Karoline Aebi-Popp, Alexia Anagnostopoulos, Manuel Battegay, Enos Bernasconi, Dominique L Braun, Heiner C Bucher, Alexandra Calmy, Matthias Cavassini, Angela Ciuffi, Günther Dollenmaier, Matthias Egger, Luigia Elzi, Jan Fehr, Jacques Fellay, Hansjakob Furrer, Christoph A Fux, Huldrych F Günthard, Anna Hachfeld, David Haerry, Barbara Hasse, Hans H Hirsch, Matthias Hoffmann, Irene Hösli, Michael Huber, David Jackson-Perry, Christian R Kahlert, Olivia Keiser, Thomas Klimkait, Roger D Kouyos, Helen Kovari, Katharina Kusejko, Niklaus Labhardt, Karoline Leuzinger, Begona Martinez de Tejada, Catia Marzolini, Karin J Metzner, Nicolas Müller, Johannes Nemeth, Dunja Nicca, Julia Notter, Paolo Paioni, Giuseppe Pantaleo, Matthieu Perreau, Andri Rauch, Luisa Salazar-Vizcaya, Patrick Schmid, Roberto Speck, Marcel Stöckle, Philip Tarr, Alexandra Trkola, Gilles Wandeler, Maja Weisser, Sabine Yerly

**Affiliations:** Department of Infectious Diseases and Hospital Epidemiology, University Hospital Zurich, 8091 Zurich, Switzerland; Institute of Medical Virology, University of Zurich, 8057 Zurich, Switzerland; Department of Infectious Diseases and Hospital Epidemiology, University Hospital Zurich, 8091 Zurich, Switzerland; Institute of Medical Virology, University of Zurich, 8057 Zurich, Switzerland; Division of Infectious Diseases, Lausanne University Hospital and University of Lausanne, 1011 Lausanne, Switzerland; Division of Infectious Diseases, Lausanne University Hospital and University of Lausanne, 1011 Lausanne, Switzerland; Department of Infectious Diseases and Hospital Epidemiology, University Hospital Zurich, 8091 Zurich, Switzerland; Department of Infectious Diseases and Hospital Epidemiology, University Hospital Zurich, 8091 Zurich, Switzerland; Institute of Medical Virology, University of Zurich, 8057 Zurich, Switzerland; Division of Infectious Diseases, Lausanne University Hospital and University of Lausanne, 1011 Lausanne, Switzerland; Global Health Institute, School of Life Sciences, Ecole Polytechnique Fédérale de Lausanne, 1015 Lausanne, Switzerland; Centre interdisciplinaire de recherche en biologie, Collége de France, 75231 Paris, France; Department of Infectious Diseases and Hospital Epidemiology, University Hospital Zurich, 8091 Zurich, Switzerland; Institute of Medical Virology, University of Zurich, 8057 Zurich, Switzerland; Department of Infectious Diseases and Hospital Epidemiology, University Hospital Zurich, 8091 Zurich, Switzerland; Department of Infectious Diseases and Hospital Epidemiology, University Hospital Zurich, 8091 Zurich, Switzerland; Institute of Medical Virology, University of Zurich, 8057 Zurich, Switzerland; Department of Infectious Diseases and Hospital Epidemiology, University Hospital Zurich, 8091 Zurich, Switzerland; Institute of Medical Virology, University of Zurich, 8057 Zurich, Switzerland; Division of Infectious Diseases, Ente Ospedaliero Cantonale, 6500 Lugano, Switzerland; Division of Infectious Diseases, University of Geneva and University of Southern Switzerland, 6900 Lugano, Switzerland; Institute of Medical Virology, University of Zurich, 8057 Zurich, Switzerland; Center for Infectious Diseases, Klinik im Park, 8027 Zurich, Switzerland; Division Infection Diagnostics, Department Biomedicine, University of Basel, 4001 Basel Switzerland; Division of Infectious Diseases and Hospital Epidemiology, University Hospital Basel, 4031 Basel, Switzerland; Division of Infectious Diseases and Hospital Epidemiology, Cantonal Hospital St. Gallen, 9007 St. Gallen, Switzerland; Division of Immunology and Allergy, Lausanne University Hospital and University of Lausanne, 1011 Lausanne, Switzerland; Department of Infectious Diseases, Inselspital, Bern University Hospital, University of Bern, 3010 Bern, Switzerland; Institute for Infectious Diseases and Multidisciplinary Center for Infectious Diseases, University of Bern, 3012 Bern, Switzerland; Division of Infectious Diseases and Hospital Epidemiology, University Hospital Basel, 4031 Basel, Switzerland; Laboratory of Virology and Division of Infectious Diseases, Geneva University Hospital, University of Geneva, 1205 Geneva, Switzerland; Department of Infectious Diseases and Hospital Epidemiology, University Hospital Zurich, 8091 Zurich, Switzerland; Institute of Medical Virology, University of Zurich, 8057 Zurich, Switzerland; Department of Infectious Diseases and Hospital Epidemiology, University Hospital Zurich, 8091 Zurich, Switzerland; Institute of Medical Virology, University of Zurich, 8057 Zurich, Switzerland

**Keywords:** HIV, neurocognitive complaints, genome-wide association study, cohort study, viral genome

## Abstract

People with HIV may report neurocognitive complaints, with or without associated neurocognitive impairment, varying between individuals and populations. While the HIV genome could play a major role, large systematic viral genome-wide screens to date are lacking. The Swiss HIV Cohort Study biannually enquires neurocognitive complaints. We quantified broad-sense heritability estimates using partial ‘pol’ sequences from the Swiss HIV Cohort Study resistance database and performed a viral near full-length genome-wide association study for the longitudinal area under the curve of neurocognitive complaints. We performed all analysis (i) restricted to HIV Subtype B and (ii) including all HIV subtypes. From 8547 people with HIV with neurocognitive complaints, we obtained 6966 partial ‘pol’ sequences and 2334 near full-length HIV sequences. Broad-sense heritability estimates for presence of memory loss complaints ranged between 1% and 17% (Subtype B restricted 1–22%) and increased with the stringency of the phylogenetic distance thresholds. The genome-wide association study revealed one amino acid (Env L641E), after adjusting for multiple testing, positively associated with memory loss complaints (*P* = 4.3 * 10^−6^). Other identified mutations, while insignificant after adjusting for multiple testing, were reported in other smaller studies (Tat T64N, Env *291S). We present the first HIV genome-wide association study analysis of neurocognitive complaints and report a first estimate for the heritability of neurocognitive complaints through HIV. Moreover, we could identify one mutation significantly associated with the presence of memory loss complaints. Our findings indicate that neurocognitive complaints are polygenetic and highlight advantages of a whole genome approach for pathogenicity determination.

## Teaser

Zeeb *et al*. report that self-reported neurocognitive complaints in people with HIV are in part heritable through the HIV genome, but in a viral genome-wide associations study, they found only one significant amino acid substitution. They conclude that self-reported neurocognitive complaints are a highly polygenic phenotype.

## Graphical Abstract

**Graphical Abstract:**
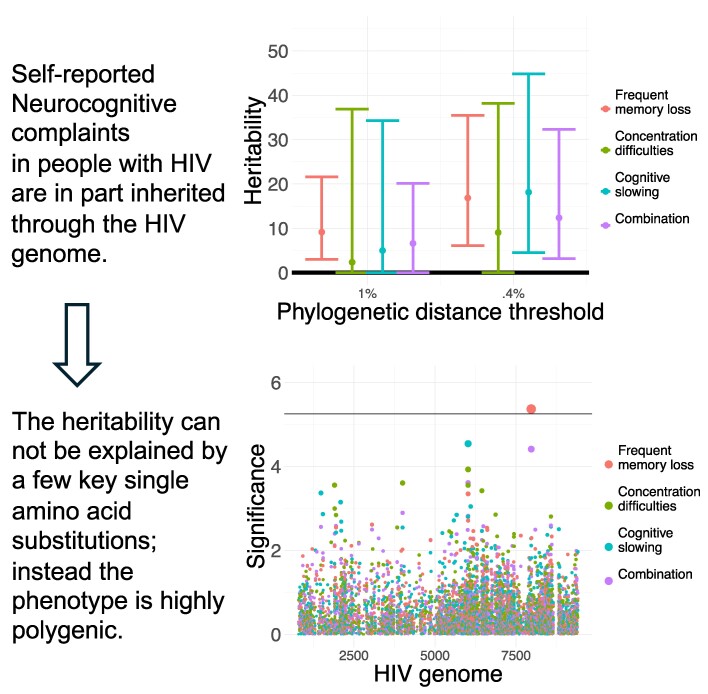

## Introduction

People with human immunodeficiency virus Type 1 (PWH) are at risk for HIV-associated neurocognitive impairments.^1-3^ Although the prevalence depends on the chosen measure, HIV-associated neurocognitive disorder (HAND) ranges from 37.4% to 52.7%, which is historically the most commonly used classification although it was shown to overestimate impairment prevalence overall and specifically those associated with HIV.^4-8^ Notably, over the >40-year course of the HIV-1 pandemic, the severity of impairments decreased.^8-11^ This effect might in part be thanks to early anti-retroviral treatment regardless of CD4+ T cell count, as viral suppression decreases inflammation and thereby the effect of HIV on neurocognitive impairment.^11-17^

Neurological impairments in PWH may have multifactorial causes, including the natural aging processes, (non-)communicable diseases, medication intake, human genetics or HIV infection.^18-20^ Nevertheless, many studies showed mechanisms of how HIV infiltrates the brain and causes neurocognitive impairments.^3,21-24^ Although there is no conclusive evidence, the current understanding is that in PWH the virus enters the brain via infected undifferentiated monocytes or T cells (Trojan horse theory).^21,25^ Once there, viruses may replicate and infect microglia cells and potentially astrocytes.^3,22,26^ Following brain infiltration, Tat is suspected to initiate an apoptotic feedback loop of neural cells, while gp120 induces the release of inflammatory cytokines causing apoptosis in neural cells.^23,24^

It has been demonstrated various times that the viral set point of PWH is a heritable viral trait, and thus, in analogy it can be inferred that different viral genotypes also express differential effects on neurocognitive impairments.^27-29^ Due to their pathophysiology, an impact of viral genotype on impairments is likely. Accordingly, HAND-associated genomic signatures were previously shown in the envelope protein (Env).^30-32^ However, so far most viral pathogenesis studies that found genetic associations were based on small sample sizes of PWH and considered HIV-associated dementia as outcome, while most PWH under antiretroviral therapy (ART) are affected by milder manifestations of HIV-associated neurocognitive impairments and/or self-reported neurocognitive complaints (SRNCs).^8,10,30,31^ However, no HIV whole genome-wide screens for these outcomes have been conducted. Since assessment of neurocognitive domain functions (e.g. required for HAND) is highly costly and time-consuming, SRNCs are an alternative for large-scale studies and were also recommended as practical approach in the clinical HIV management.^1,8,10,20,33^ The use of this approach is also supported by previous findings, that SRNCs are associated with HIV phylogenetic clusters.^34^

The Swiss HIV Cohort Study (SHCS) provides a unique opportunity to study longitudinal SRNCs in PWH and matching viral sequencing data. Based on these comprehensive data, we performed a systematic assessment of the association between viral genotypes and SRNCs: specifically, we first estimated their viral heritability to justify further genomic analyses and the effect of subtype and performed the first-of-its-kind viral genome-wide association study (GWAS) for SRNCs.

## Materials and methods

### Study population and ethics

Our study population includes PWH enrolled in the SHCS. The SHCS is a multicentre, open-label, non-randomized, longitudinal, prospective cohort study in Switzerland, which has recruited almost 22 000 PWH since 1988.^35^

The SHCS was approved by the ethics committees of the participating institutions (Kantonale Ethikkommission Bern, Ethikkommission des Kantons St. Gallen, Comité Départemental d’Éthique des Spécialités Médicales et de Médicine Communataire et de Premier Recours, Kantonale Ethikkommission Zürich, Repubblica et Cantone Ticino–Comitato Ethico Cantonale, Commission Cantonale d’Éthique de la Recherche sur l’Être Humain, Ethikkommission beider Basel for the SHCS and Kantonale Ethikkommission Zürich for the Zurich Primary HIV Infection Cohort Study), and written informed consent was obtained from all participants.

### Phenotype definition

In the SHCS, SRNCs have been systematically assessed biannually since 2013 via questionnaire, according to the European clinical AIDS guidelines, i.e. by asking the SHCS participants: ‘Is the patient aware of frequent memory loss in daily life?’ (frequent memory loss), ‘Does the patient experience difficulties in paying attention in normal daily life?’ (concentration difficulties) and ‘Is the patient aware of slowing down in reasoning or solving problems?’ (cognitive slowing).^1,36^ The three possible answers for each question were ‘never’, ‘hardly ever’ or ‘yes, definitely’. Allowing for a lag time until full implementation of the questionnaire in all centres and a potential bias introduced due to the SARS-CoV-2 pandemic, we considered questionnaires conducted between 2014 and 2020. To ascertain robust time trends from individual PWH and to account for extreme measures at single time points, we restrained our analysis to participants with at least five answered questionnaires. We assigned a numeric score (0, 1 and 2) to the three possible answers for each question and calculated the area under the curve (AUC) divided by the follow-up time for all question-scores separate (i.e. frequent memory loss, concentration difficulties and cognitive slowing) and the combination, as previously described.^10^ We considered the AUCs as a continuous outcome for tobit regression-based analyses (given the large number of participants with AUC = 0). Since HIV-1 Subtype B is predominant in Switzerland and to avoid potential noise from non-B subtypes, we performed all analyses with all subtypes included and restricted to Subtype B (for subtype determination see supplementary sensitivity analysis—subtype).

### Definition of confounders and comorbidities

We adjusted analyses for several characteristics of PWH (Table 1 and  Supplementary Table 1).

**Table 1:** Characteristics of study participants overall and stratified by HIV-1 subtype B versus non-B

	Overall	HIV subtype B	Subtype non-B	*P*
*n*	8547	5815	2732	
Female, *n* (%)	2491 (29.1)	1113 (19.1)	1378 (50.4)	<0.001
SHCS enrolment year, median (IQR)	2004 (1997, 2009)	2002 (1996, 2009)	2006 (2001, 2011)	<0.001
Age at first SRNC questioning, median (IQR)	48 (40, 54)	49 (42, 54)	44 (37, 52)	<0.001
Timeframe of reported SRNCs in years, median (IQR)	5.12 (4.79, 5.42)	5.13 (4.81, 5.43)	5.09 (4.73, 5.41)	<0.001
Number of study visits with SRNC questionnaire, *n* (%)				<0.001
5–7	1553 (18.2)	972 (16.7)	581 (21.3)	
8–10	5621 (65.8)	3863 (66.4)	1758 (64.3)	
11–13	1373 (16.1)	980 (16.9)	393 (14.4)	
Complaint combination (AUC), median (IQR)	0.04 (0.0, 0.25)	0.04 (0.0, 0.25)	0.04 (0.0, 0.24)	0.329
Frequent memory loss (AUC), median (IQR)	0.06 (0.0, 0.40)	0.06 (0.0, 0.41)	0.05 (0.0, 0.39)	0.329
Concentration difficulties (AUC), median (IQR)	0.0 (0.0, 0.23)	0.00 (0.0, 0.24)	0.0 (0.0, 0.22)	0.056
Cognitive slowing (AUC), median (IQR)	0.0 (0.0, 0.10)	0.00 (0.0, 0.10)	0.0 (0.0, 0.10)	0.359
Education, *n* (%)				<0.001
None	572 (6.7)	221 (3.8)	351 (12.8)	
Mandatory school	1387 (16.2)	784 (13.5)	603 (22.1)	
Higher education	6323 (74.0)	4586 (78.9)	1737 (63.6)	
Other	265 (3.1)	224 (3.9)	41 (1.5)	
Mode of HIV-1 acquisition, *n* (%)				<0.001
HET	3300 (38.6)	1387 (23.9)	1913 (70.0)	
MSM	3811 (44.6)	3314 (57.0)	497 (18.2)	
Other	1436 (16.8)	1114 (19.2)	322 (11.8)	
HIV-1 RNA viral load (log10; AUC), median (IQR)	1.35 (0.55, 2.28)	1.44 (0.63, 2.33)	1.13 (0.43, 2.18)	<0.001
CD4+ T cell count (AUC), median (IQR)	483.3 (362.6, 615.0)	488.2 (370.3, 619.5)	474.2 (348.3, 603.4)	<0.001
Ethnicity, *n* (%)				<0.001
White	6663 (78.0)	5334 (91.7)	1329 (48.6)	
Black	1228 (14.4)	117 (2.0)	1111 (40.7)	
Hispano-American	275 (3.2)	215 (3.7)	60 (2.2)	
Other	381 (4.5)	149 (2.6)	232 (8.5)	
Time of efavirenz use in years, mean (SD)	0.08 (0.76)	0.06 (0.70)	0.11 (0.86)	0.011
Any history of antidepressants use, *n* (%)	1537 (18.0)	1178 (20.3)	359 (13.1)	<0.001
Any history of depression, *n* (%)	2426 (28.4)	1808 (31.1)	618 (22.6)	<0.001
Any history of drug use, *n* (%)	2246 (26.3)	1867 (32.1)	379 (13.9)	<0.001
Any neurological disease, *n* (%)	511 (6.0)	351 (6.0)	160 (5.9)	0.781
Hepatitis C, *n* (%)	1425 (16.7)	1202 (20.7)	223 (8.2)	<0.001
Hepatitis B, *n* (%)	2159 (25.3)	1465 (25.2)	694 (25.4)	0.856

Abbreviation: HET, heterosexual; IQR, interquartile range; MSM, men who have sex with men.

We adjusted for HIV-1 population structure using principal components analysis (PCA) estimated with the implementation of Eigensoft with iterative outlier removal on nucleotide level for each gene/protein.^37,38^ For this we binarized multiallelic sites (major versus each minor variant). We adjusted for the first 10 PCAs, calculated on the respective gene/protein.

### Sequencing

We used two distinct sequence databases: (i) an Next Generation Sequencing (NGS) database with near full-length HIV-1 genome sequences obtained from long overlapping amplicon sequencing on Illumina MiSeq.^39,40^ We included samples from plasma virions (54.2%) and proviral origin (45.8%) from various sampling time points (Supplementary Table 2). All sequences were originally sequenced for purposes unrelated to this study. Furthermore, some study individuals have genes from different near full-length sequence samples (Supplementary Table 3). (ii) a partial ‘pol’ region sequence database, primarily maintained for routine genotypic HIV drug resistance testing. PWH in the SHCS are tested for drug resistance, at time of entering the SHCS if not virally suppressed, or if treatment failure occurs. More than 11 000 sequences also have been retrospectively generated from samples stored in the SHCS biobank.^41^ For the NGS data set, we assembled reads from Illumina MiSeq with an in-house sequence alignment tool (available at https://github.com/medvir/SmaltAlign). Alignment was done with an initial alignment to the HIV-1 reference genome HXB2 including *de novo* assembled sequences, followed by three alignments against iteratively improved references.^42^ From the final sequence alignment, we generated the majority consensus sequence with a depth threshold of ≥20 for each position. We extracted the respective nucleotide regions from each sequence using the local version of NCBI BLAST.^43^ The BLAST database consisted the appropriate regions from a panel of 459 reference sequences obtained from the Los Alamos HIV sequence database (https://www.hiv.lanl.gov/). We made codon alignments of all blasted gene sequences using MACSE v2, to account for frameshifts, for the amino acid translation.^44^ For each gene region on amino acid level, we generated multiple sequence alignments (MSAs) using Mafft.^45^ Prior MSA, we removed sequences with a coverage of less than 40% of HIV-1 HXB2 of the respective genome. We generated nucleotide MSAs with reverse translation of the MSAs on amino acid level to the original nucleotide sequence.

### Heritability

We estimated a maximum likelihood phylogeny based on partial ‘pol’ sequences using IQtree2 and extracted clusters based on different phylogenetic distance thresholds (5%–0.4%).^46^ We included the obtained clusters in a mixed-effect tobit regression model implemented in STATA adjusted for a range of covariables (Supplementary Table 1). We quantified the broad-sense heritability and its 95% confidence interval by calculating the intraclass correlation, i.e. the within-cluster correlation, based on similar approaches used for the determination of HIV-1 heritability.^27,47,48^

### Genome-wide association study

We set the minor amino acid frequency threshold at 60 and the minimum frequency for the reference to 300. We adjusted for multiple testing by Bonferroni correction based on effective test size, which adjusts for linkage disequilibrium. Effective test size was computed as the number of eigenvalues needed to explain 99.5% of the variance in the association matrix, i.e. Cramér’s V between all variants.^49^ We further assessed a possible dose response of the within-sequence-nucleotide-frequency of the nucleotide responsible for the respective amino acid polymorphism. For the GWAS analysis, we used a tobit model implemented in the R package Applied Econometrics with R with a lower censoring threshold at 0. We adjusted the GWAS analyses for a range of covariables (Table 1 and  Supplementary Table 1) and population structure, i.e. the first 10 viral PCAs.^50^

### Software

We performed statistical analysis in R 4.2.1 and Stata. We acknowledged additional software in the respective sections.

We followed the Strengthening the Reporting of Observational Studies in Epidemiology reporting guidelines (STROBE).^51^

## Results

**Figure 1:**
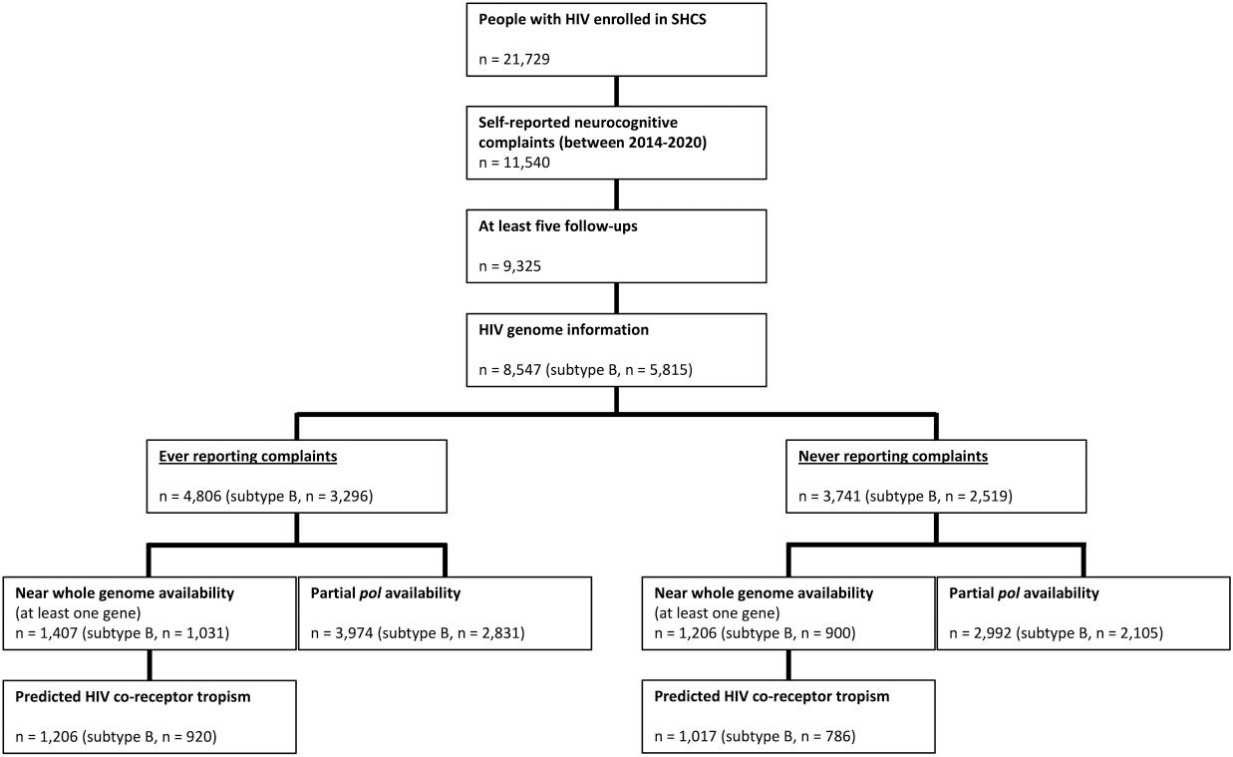
Flowchart showing available SHCS participants with and without SRNCs and HIV-1 genome data (status from 1 May 2023; Subtype distribution: see Supplementary Table 4).

**Figure 2:**
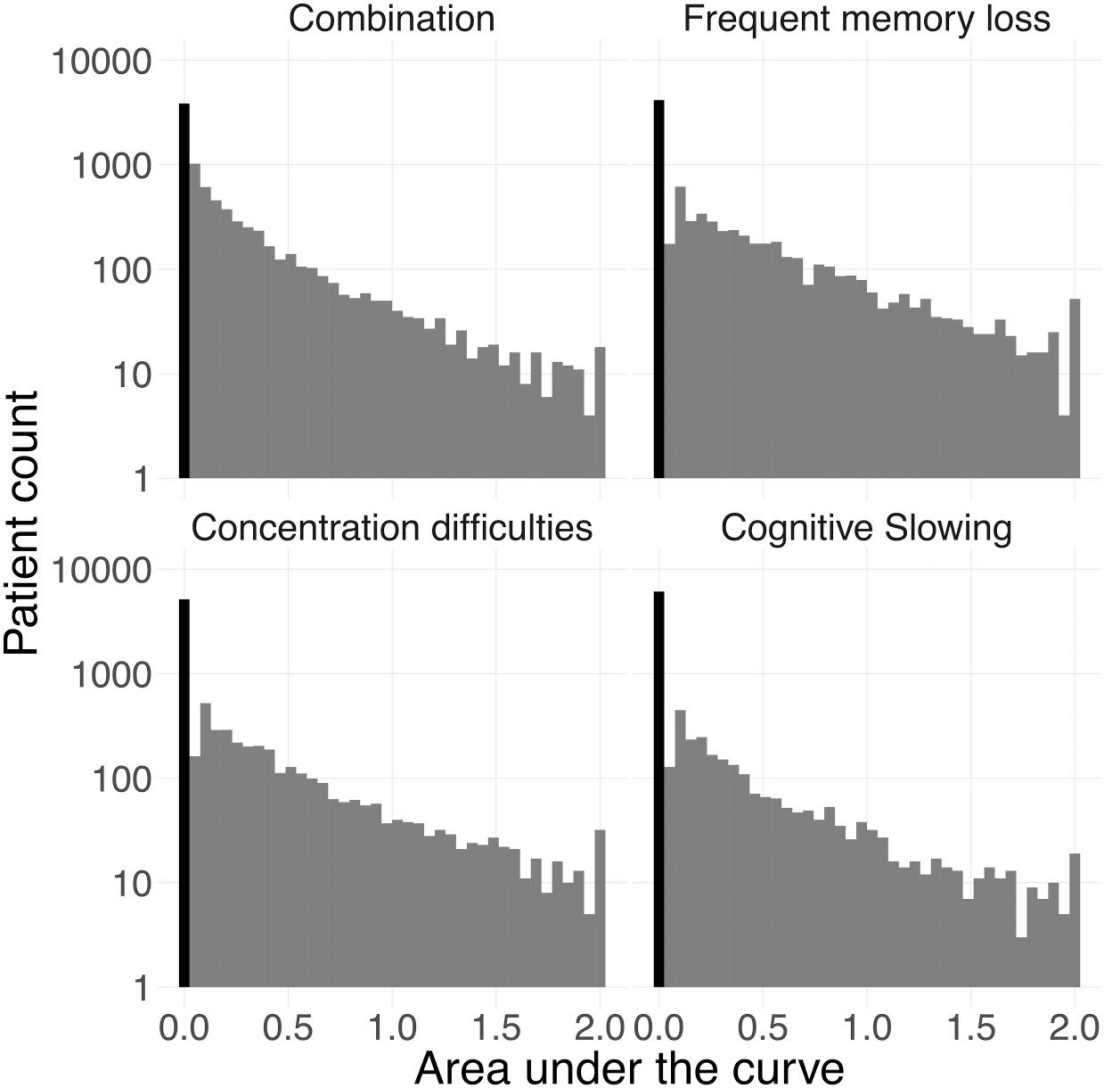
SRNCs among people with HIV (PWH) in Switzerland as the AUC across study visits in the SHCS. SRNCs are defined with the following levels: 0, ‘no’; 1, ‘hardly ever’; and 2, ‘yes, definitely’, for cognitive slowing, concentration difficulties, frequent memory loss and their combination. The bold bar indicates those with an AUC of 0, i.e. PWH who never report the respective SRNCs.

Among the 8547 out of 21 729 PWH fulfilling the inclusion criteria (Table 1), we obtained 6966 partial ‘pol’ sequences. We obtained 3287 near whole genome sequences from 2613 unique PWH. For individual proteins, the minimum was 2129 sequences for Env and the maximum 2334 sequences for Nef (Fig. 1 and Supplementary Table 3). From the selected population, a substantial proportion (43.8%) never reported any SRNCs leading to zero-inflated AUC distributions (Fig. 2). Prevalence of ever reporting SRNCs, i.e. AUC above 0, was 56.2% for their combination, 51.3% for frequent memory loss, 39.8% for concentration difficulties and 28.4% for cognitive slowing. Correlations between AUC phenotypes are highest between combination and concentration difficulties (Pearson correlation 0.95) and lowest between frequent memory loss and cognitive slowing (Pearson correlation 0.75; Supplementary Table 5).

### Self-reported neurocognitive complaints are a heritable HIV-1 trait

**Figure 3:**
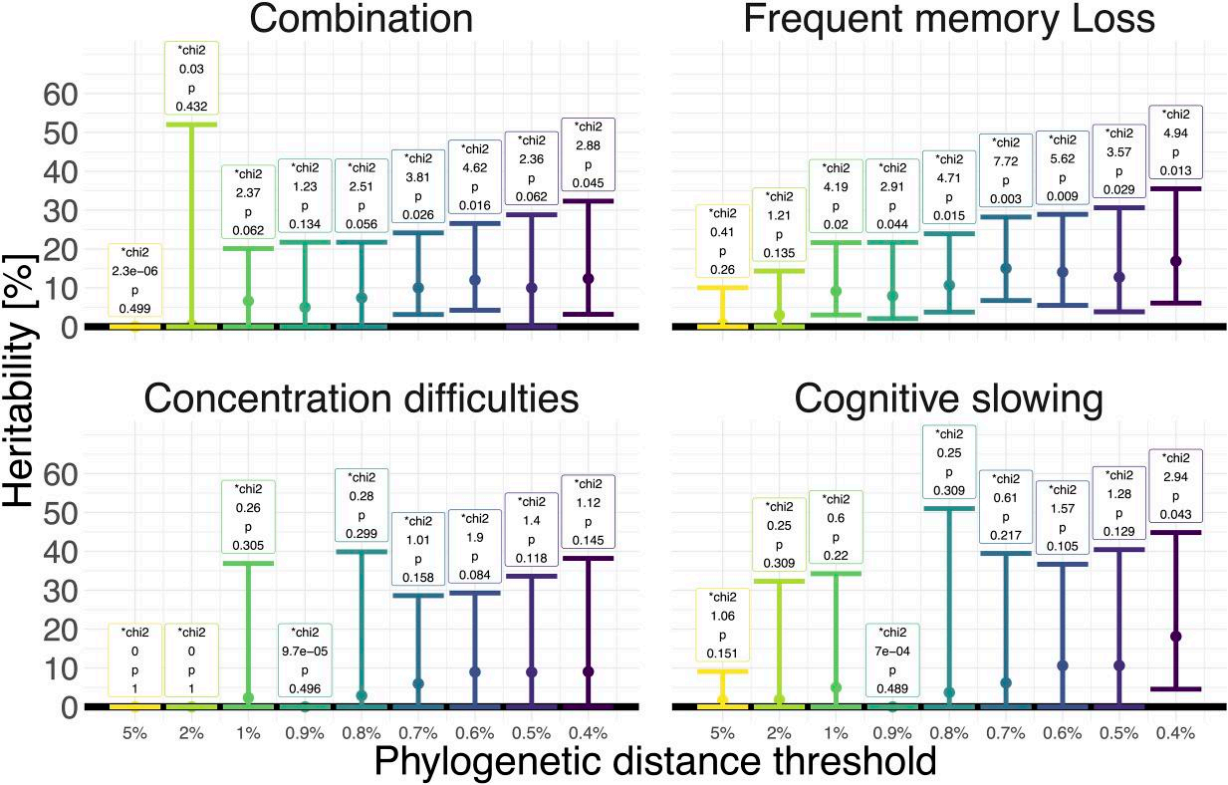
Broad-sense heritability of the SRNCs phenotypes cognitive slowing, concentration difficulties, frequent memory loss and their combination approximated by intraclass correlation (ICC). ICC is estimated by comparison of a mixed tobit model using phylogenetic cluster under specified thresholds as random effects and a tobit model without mixed effects. The number above the bars indicate the test statistic [*test statistic: χ^2^ (chi2)] and *P* value of the respective ICC. Phenotypes are calculated as the AUC of longitudinally measured cognitive slowing, concentration difficulties, frequent memory loss or the combination of all three. Analysis was done on 6966 partial ‘pol’ (all subtypes) sequences. Absence of confidence intervals is non-converged models.

We estimated the heritability of SRNCs to assess the overall genetic effects. We inferred from 6966 (all HIV-1 subtypes) and 4936 (HIV-1 Subtype B) partial ‘pol’ sequences maximum likelihood phylogenetic trees and extracted clusters under pre-defined phylogenetic distance thresholds. Using random-effect tobit models to estimate the overall fraction of variance explained by the viral genome (i.e. the broad-sense heritability, see Materials and methods), we observed significant estimates that increased with stricter distance thresholds: for frequent memory loss heritability increased from 1% (95% CI = 0%, 10%) to 17% (6%, 35%) with a phylogenetic distance threshold increase from 5–0.4% (Fig. 3). This result was even more prominent when restrained to Subtype B, where it increased respectively from 9% (2%, 24%) to 22% (9%, 42%; Supplementary Fig. 1). For the combined SRNCs, heritability increased to 12% (3%, 32%) among all subtypes and to 20% (8%, 40%) among Subtype B. For concentration difficulties, heritability increased to 9% (0%, 38%) among all subtypes and to 19% (5%, 44%) among Subtype B. For cognitive slowing, heritability increased to 18% (5%, 45%) among all subtypes and to 23% (7%, 51%) among Subtype B.

### Genome-wide association study finds significant association with Env L641E mutation

**Figure 4:**
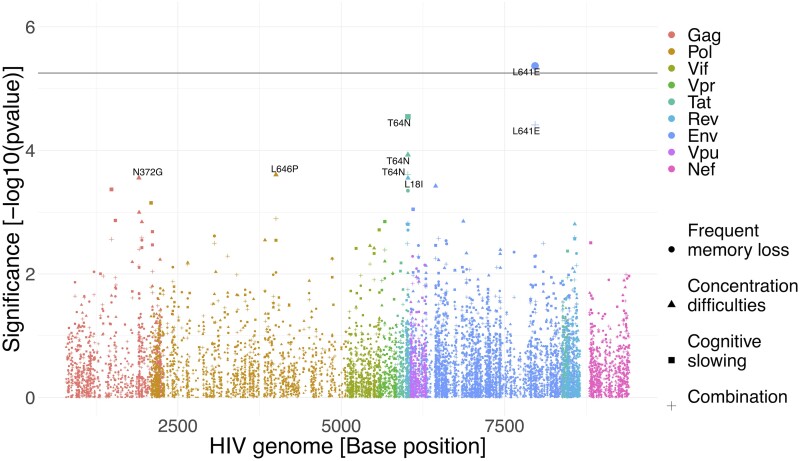
Genome-wide association study of the HIV-1 genome (including all subtypes) and associations with SRNCs in people with HIV (PWH). SRNCs are defined as the AUC of longitudinally measured cognitive slowing, concentration difficulties, frequent memory loss or the combination of all three. The *P* values were calculated with a multivariable tobit model (test statistic: *z*).

As we determined a significant heritability across phenotypes, we next assessed associations of genetic polymorphisms using a GWAS analysis at the amino acid level across the nine HIV-1 main proteins. We found several strong associations between amino acid variants and SRNCs although only one was still significant after adjusting for multiple testing (*P* < effective multiple testing threshold, 5.6 * 10^−6^). In particular, the variant Env L641E in the C-terminal heptad-repeat region was associated with more frequent memory loss AUC (*P* = 4.3 * 10^−6)^ and more combination SRNCs AUC (*P* = 3.9 * 10^−5^), while Tat T64N was associated with less cognitive slowing AUC (*P* = 2.9 * 10^−5^; Fig. 4). When restricted to Subtype B (effective multiple testing threshold 8.6 * 10^−6^), Rev L18I (*P* = 1.7 * 10^−5^), Tat T64N (*P* = 3.3 * 10^−5^) and Env T464N (*P* = 8.5 * 10^−5^) were associated with less concentration difficulties AUC (Supplementary Fig. 2). All other associations were *P* > 1.0 * 10^−4^ (Supplementary Tables 6 and 7). Quality check of GWAS models indicates well-controlled models with no *P* inflation (Supplementary Figs. 3 and 4). Several covariables did show significant associations with the outcomes. The first 10 PCAs explained between 3.5% and 11% of the viral genetic variance (Supplementary Table 8). The models including only covariables and population structure without amino acid mutations, based on the PWH population with partial ‘pol’ sequences, are shown in Supplementary Figs. 5–8 (all HIV-1 subtypes) and Supplementary Figs. 9–12 (Subtype B).

For the top amino acid mutations (Env L641E, Tat T64N, Env T464N and Rev L18I), we further tested if the effects are dependent on the nucleotide frequency. Overall, increasing frequencies showed similar trends as the actual amino acid mutations but were not statistically significant (Supplementary Table 9).

## Discussion

We performed the first large-scale HIV-1 GWAS with SRNCs in people with HIV with well-controlled HIV-1 infection. We showed that the HIV-1 genome explains a significant part of variations in SRNCs. Moreover, we found one genetic variant in the Env protein (L641E) significantly associated with frequent memory loss AUC. Several other variants are associated although not significantly after adjusting for multiple testing. However, they are biologically plausible and were previously reported for related outcomes (see below).

### Heritability

To our knowledge, estimates for heritability of SRNCs or even neurocognitive impairments on the HIV-1 genome side have not been performed. Addressing this gap, our phylogenetic analysis shows significant broad-sense heritability estimates for combination SRNCs, frequent memory loss SRNCs and cognitive slowing SRNCs AUC. These effects are stronger with stricter phylogenetic cluster distance thresholds, i.e. the more similar HIV-1 sequences within a cluster are the higher the variance explained by these clusters. This implies a substantial overall impact of the viral genotypes on SRNCs. The phylogenetic cluster approach was previously used to determine the heritability of the HIV-1 set-point-viral load and showed good overlap with alternative methods, which do not require the definition of clusters but are not adapted to zero-inflated data.^27,47,48^

### Viral genome-wide association study

The significant heritability provides the rationale for testing the impact of individual variants in a viral GWAS. The association between Env L641E and more frequent memory loss was the only significant effect after correction for multiple testing. L641E was previously reported to be correlated with Env 300 among 15 CSF-derived sequences.^52^ Env 300 was also reported to be highly predictive of impaired neurocognitive performances among 18 individuals.^53^ However, we found no correlation between Env 641 and Env 300 and no association of Env 300 with SRNCs. It is possible this correlation is only selected for in the CSF. Besides L641E, Tat T64N shows altogether the strongest signal across all phenotypes, especially negative associations with concentration difficulties and cognitive slowing AUC. An increased frequency of T64N was reported in the CSF, CNS and lymphoid tissue of PWH deceased with HIV-associated dementia or encephalitis.^54^ It was shown that T64 increases Tat phosphorylation, which increases binding to the transactivation response element and subsequent viral replication.^55^ In contrast T64N decreases viral replication, therefore potentially decreasing SRNCs. Further, Rev L18I is negatively associated with concentration difficulties AUC. However, this may be a side effect of Tat T64N, which requires the same nucleotide change. Last, Env T464N (HXB2: E464) in the V5 loop in a glycosylation site is negatively associated with concentration difficulties AUC. This glycosylation site, although specifically the Position 463, was reported to increase neutralization sensitivity to the broadly neutralizing antibody VRC01 and was also shown to be a HAND-associated hotspot.^52,56^ The introduction of N464 might similarly increase sensitivity of adaptive immune responses due to changes in HIV glycosylation. Despite these associations, our results suggest a discrepancy between the substantial heritability and the relatively weak effect of individual variants. This was previously termed as ‘the missing heritability’, indicating a high polygenicity of SRNCs.^57^ Accordingly, we explored in more detail the effect of viral subtype and co-receptor but found no consistent effects (see supplementary sensitivity analysis—Subtype/Tropism, Supplementary Figs. 13–16). Finally, as an overall validation of the top associated mutations, we performed cross validation that indicated increased explained variance in models with genotypes included compared with models with only patient characteristics and population structure included (see supplementary sensitivity analysis—Cross validation, Supplementary Fig. 17).

### Mixed comparability with previous found amino acid substitutions

Comparing our GWAS results at a nominal significance level of *P* ≤ 0.05 to studies reporting HAND-associated amino acid polymorphisms reveals some overlap but also contradictions (Supplementary Table 10). Our results are aligned with the observed negative association between Env *291S and HAND.^31^ Similarly, we found positive associations for all SRNC AUCs with Env E293K, which was more frequently observed in CSF-derived compared with plasma-derived sequences.^58^ A positive HAND association was reported for Env R315K, whereas we observe a negative association with concentration difficulties AUC.^30,31^ A similar contradiction was obtained for Env *340N, which was reported as protective for HAND, whereas we found a positive association with frequent memory loss AUC.^31^ Finally, Env *308H was reported as a signature of CSF-derived sequences compared with plasma-derived sequences, while we observed negative associations with all phenotypes.^58^ These contradictions may reflect differences in the sample types, outcomes and study design. First, all mentioned studies included CNS- or CSF-derived sequences, whereas we focus on plasma-derived sequences, although Ogishi and Yotsuyanagi^31^ showed that HAND-associated signatures were shared among all sample sources (CNS, CSF, lymphatic system and peripheral circulation). Secondly, Holman *et al.*^30^ and Ogishi and Yotsuyanagi^31^ compared PWH with and without HIV-associated dementia, whereas we considered SRNCs covering mostly asymptomatic to moderate outcomes. Thirdly, these studies included below 100 unique PWH and did not perform a whole genome approach.^30,31^ Finally, differences between identified mutations here and reported signature CSF mutations are expected, as the latter are not necessarily linked to SRNCs or HAND but may just represent general adaptations to the brain environment.^58^

### Limitations

Our study has several limitations. In the SHCS, PWH rarely experience severe cognitive impairments, meaning they have no impact on their daily activities.^5^ In comparison, SRNC prevalence (56%) is higher than the neurocognitive impairments prevalence (32%) in Switzerland.^5^ Phenotypic differences are also shown by the low predictive value of SRNCs for HAND.^59^ Together this indicates that (i) a fraction of SRNCs might be non-HIV-1 related and (ii) associations between HIV-1 and neurocognitive impairments are underestimated by SRNCs, i.e. an absence of SRNCs does not imply an absence of HIV-associated impairments. This makes it challenging to assess the effect of genetic variants on neurocognitive impairment. As a result, we might have underestimated the viral genetic impact. Moreover, we included both plasma- and proviral-derived sequences sampled at different time points to increase statistical power, but this might have diluted the results. On the other hand, we still found plausible amino acid mutations and significant broad-sense heritability estimates across phenotypes. Further, we adjusted for potential confounding factors and comorbidities to minimize bias and performed cross validation. Hence, our results are unlikely to be due to confounding although we cannot exclude unobserved confounders. Finally, only adjusting for population structure omitting covariables yields similar GWAS results, suggesting only minor confounding of these covariables (Supplementary Figs. 18 and 19).

Variation in the result overlaps compared with other studies and also overall variation depending on the genomic model and outcome confirm the high diversity of SRNCs and impairments. A single outcome definition, be it SRNCs or HAND, is likely insufficient to reflect clinical varieties. While HAND is without a doubt a better measurement than SRNCs, it still reduces a multitude of measures of different cognitive domains into one diagnosis, with arbitrary cut-offs, which overestimates the prevalence of neurocognitive impairments.^7,20^ In particular, the Frascati criteria for HAND are very strict and should be replaced by a more flexible measure such as *z*-/*t*-scores based on neurocognitive domain function, as already done by Wang *et al*.,^6,60^ or a more qualitative approach as proposed by Nightingale *et al*.^8^ in combination with temporally fitting NGS sequences. Such measures will allow to better capture the biological mechanisms due to diverse genetic effects (human or viral).

## Conclusion

In this large phylogenetic and GWAS analysis, we found a substantial and significant heritability of SRNCs in PWH and could identify one viral variant that was significantly associated with SRNCs and several more candidates, which were nominally significant though not after adjusting for multiple testing but were previously linked to HIV-associated dementia or CSF-derived sequences. Our work adds to the growing evidence for the impact of viral genomic variation on infectious disease pathogenicity.

## Supplementary Material

fcae188_Supplementary_Data

## Data Availability

The underlying code for the analyses is available at https://github.com/M-Zeeb/HIV_neuro_GWAS. The individual-level data sets generated or analysed during the current study do not fulfil the requirements for open data access: (i) the SHCS informed consent states that sharing data outside the SHCS network is only permitted for specific studies on HIV infection and its complications and to researchers who have signed an agreement detailing the use of the data and biological samples, and (ii) the data are too dense and comprehensive to preserve patient privacy in persons living with HIV. According to the Swiss law, data cannot be shared if data subjects have not agreed or data are too sensitive to share. Investigators with a request for selected data should send a proposal to the respective SHCS address (www.shcs.ch/contact). The provision of data will be considered by the Scientific Board of the SHCS and the study team, is subject to Swiss legal and ethical regulations and is outlined in a material and data transfer agreement.

## Appendix

### Members of the Swiss HIV Cohort Study

Irene Abela, Karoline Aebi-Popp, Alexia Anagnostopoulos, Manuel Battegay, Enos Bernasconi, Dominique L. Braun, Heiner C. Bucher, Alexandra Calmy, Matthias Cavassini, Angela Ciuffi, Günther Dollenmaier, Matthias Egger, Luigia Elzi, Jan Fehr, Jacques Fellay, Hansjakob Furrer, Christoph A. Fux, Huldrych F. Günthard, Anna Hachfeld, David Haerry, Barbara Hasse, Hans H. Hirsch, Matthias Hoffmann, Irene Hösli, Michael Huber, David Jackson-Perry, Christian R. Kahlert, Olivia Keiser, Thomas Klimkait, Roger D. Kouyos, Helen Kovari, Katharina Kusejko, Niklaus Labhardt, Karoline Leuzinger, Begona Martinez de Tejada, Catia Marzolini, Karin J. Metzner, Nicolas Müller, Johannes Nemeth, Dunja Nicca, Julia Notter, Paolo Paioni, Giuseppe Pantaleo, Matthieu Perreau, Andri Rauch, Luisa Salazar-Vizcaya, Patrick Schmid, Roberto Speck, Marcel Stöckle, Philip Tarr, Alexandra Trkola, Gilles Wandeler, Maja Weisser and Sabine Yerly.
