# Supplementary material for: Self-reported neurocognitive complaints in the Swiss HIV Cohort Study: a viral genome-wide association study
Source: Brain Commun. 2024 May 31;6(4):fcae188. doi: 10.1093/braincomms/fcae188 (PMC11220509; doi:10.1093/braincomms/fcae188)
Supplement: fcae188_Supplementary_Data [file fcae188_supplementary_data.docx]

**Supplementary material**

**Sensitivity analysis - Subtype/Tropism**

**Methods - Sensitivity analysis - Subtype/Tropism**

**HIV-1 subtype and co-receptor tropism determination**

We determined the HIV-1 subtype for each individual from near whole genome sequences using comet.^1^ In case of multiple samples assigned to different subtypes, we used the subtype with highest certainty. If near whole genome was unavailable or the subtype unassigned by comet, we determined it from partial *pol* sequences using rega.^2^ If both near whole genome and partial *pol* sequences had unassigned subtypes, we used the subtype from geno2pheno.^3^ If no subtype from any method was assigned, we classified the individual as subtype “others”. We assessed the association of SRNCs with co-receptor tropism (CXCR4 vs. CCR5) inferred by geno2pheno.^3^ Geno2pheno reports prediction in the form of a false positive rate (FPR): a value of 0% indicates CXCR4 capability whereas 100% indicates CXCR4 inability. Since for most samples the FPR is not exactly 0% or 100% we used a previously defined FPR threshold to categorize tropism based on the MOTIVATE trials^4^: CCR5 only (FPR >5.75%) or CXCR4 capable (FPR ≤5.75%). In case of multiple sequences giving different results, we took the first sample according to sample date.

**HIV-1 subtype and co-receptor tropism analyses**

We assessed SRNCs differences between HIV-1 subtype B, subtypes with n>40 (AG, A, AE, D, C, F, G), and subtypes n<=40 combined as “others”. We used likelihood ratio test between an empty model and the model with the subtypes included. If the likelihood ratio test was significant (p≤0.05) we assessed the differences of individual subtypes compared to subtype B. We implemented this comparison in the whole population, and in the subgroup of participants of White ethnicity. We assessed SRNCs differences between all three defined HIV-1 tropisms. For both tropism and subtype analyses we used a univariable tobit model implemented in the R package AER with a lower censoring threshold at 0.^5^

**Results - Sensitivity analysis - Subtype/Tropism**

**HIV-1 subtype and CXCR4 tropism positively associated with SRNC**

Since the heritability estimates are not explained by single amino acid substitutions, we assessed the impact of more coarse-grained genomic factors. HIV-1 subtype is known to be associated with neurocognitive impairments (e.g., subtypes G, D, A) (study subtype distribution: Supplementary Table 4). We found an association between HIV-1 subtype and cognitive slowing AUC (likelihood ratio test p=0.05) including PWH of all ethnicities. Closer inspection reveals a positive association between cognitive slowing AUC and subtypes G (0.18, (0.04, 0.33)) and D (0.22, (0.01, 0.42)) compared to subtype B when including PWH participants of all ethnicities (Supplementary Figure 13). However, we found no significant association between cognitive slowing AUC and HIV-1 subtype when restricting our analysis to participants of White ethnicity (Supplementary Figure 14).

Next, we assessed HIV co-receptor tropism from 2,223 near whole genome sequences successfully genotyped by geno2pheno, 1,907 were classified as CCR5 tropic only and 316 as CXCR4 tropic. CXCR4 tropism was positively associated with concentration difficulties AUC (coefficient = 0.13 (0.003, 0.25)) and cognitive slowing AUC (coefficient = 0.15 (0.02, 0.29)) compared to CCR5 tropism in the analysis restricted to subtype B (all subtypes: Supplementary Figure 15; subtype B: Supplementary Figure 16). As tropism might be confounded by the duration of infection, we performed sensitivity analyses adjusting for time since infection (approximated by average pairwise diversity (APD) ^6^) and co-receptor tropism in PWH with sequence sampling before ART initiation. In the adjusted analyses, the previously observed associations between concentration difficulties-/cognitive slowing AUC and co-receptor tropism became insignificant (subtype B: concentration difficulties AUC: coefficient = 0.06 (-0.17, 0.29); cognitive slowing AUC: coefficient = 0.17 (-0.08, 0.43)).

**Discussion - Sensitivity analysis - Subtype/Tropism**

**Subtype D and G positively associated with SRNC**

As heritability estimates might be partly driven by HIV-1 subtypes, we determined SRNC AUC differences between subtype- B and -non-B. This yielded two positively associated subtypes (A and G) compared to B. The positive association of cognitive slowing AUC with subtype D is in line with previous studies. PWH with subtype D are known to experience faster disease progression and mortality.^7^ Moreover, a high proportion of dementia was observed among PWH in Uganda with subtype D compared to A.^8^ However, a subsequent study in Ugandan children found the opposite effect.^9^ Possible explanations are differences in disease advancement and the gene used for subtyping. Both studies showed the strongest effects based on *env* subtyping, whereas we predominantly used partial *pol* sequences. Therefore, and because of geographical setting differences, a comparison is limited, although our study population is likely more comparable to Sacktor et al. than Biovine et al. The subtype G effect is in line with decreased cognitive performance in treatment-naive PWH in Nigeria with subtype G compared to CRF02_AG.^10^

**CXCR4 tropism positively associated with SRNC**

Another potential driver of phenotypic differences is the usage of HIV-1 co-receptor tropism. We found that PWH with a CXCR4 tropic virus report more SRNCs than PWH with a CCR5 only capable virus among PWH with subtype B. In contrast, many studies show CCR5 as the most important for brain infiltration and neurocognitive impairments.^11–14^ However, CXCR4 was also linked to impairments: a study in mouse models found that gp120 induced aberrant, rod-shaped cofilin-actin inclusions which disrupted normal brain activity more potently via CXCR4 than CCR5.^15^ Moreover, Morris *et al.*^16^ showed increased HAND during acute infection and dual tropic viruses. It is known that CCR5, primarily on macrophages, is important in early infection and CXCR4, primarily on T cells, in advanced infection.^17^ Therefore, CXCR4 sequences were possibly from later timepoints in the infection with longer time before ART initiation. The adjusted analysis with APD as proxy for time since infection confirms this, which shows loss of significance. Therefore, confounding due to time since infection is likely the explanation of the CXCR4 effects, especially since we only assessed genotypes from plasma. However, considering other research, true CXCR4 driven mechanisms cannot be excluded.

**Sensitivity analysis – Cross validation**

**Methods - Sensitivity analysis – Cross validation**

We performed a cross validation to validate the top amino acid substitutions found in GWAS for each respective phenotype and subtype as follows:

1. We performed 4000 bootstraps each for every possible combination of the following factors: Subtype (B and all), phenotype (memory loss, concentration difficulties, cognitive slowing, and the combination), and top significant amino acid substitutions from GWAS for each respective phenotype (top 5, top 10, and top 20).
2. In each bootstrap we split 80% of the data into a training dataset and the other 20% into a testing dataset.
3. In the training dataset we performed 10fold lasso cross validation implemented in the R package tobitnet by Jacobson, T. and Zou, H.^18^ Once with a full model, i.e., covariables and the top amino acid substitutions from the respective GWAS as predictors and once with a partial model, i.e., only with the covariables.
4. Based on the best models of step 3 we predicted the phenotype in the test dataset and computed the squared pearson correlation (R^2^) between the prediction and the true phenotype. As required for tobit regression, we set negative predictions as 0 (lower censoring bound).
5. Finally, we calculated the R^2^ difference between the full model and the partial model.
6. We report the distribution of the differences of the R^2^ over all 4000 bootstraps from both models.

**Results - Sensitivity analysis – Cross validation**

The median differences between the full and partial models are between 0.0037 (all subtypes, cognitive slowing, top 5 amino acid substitutions) and 0.044, (all subtypes, concentration difficulties, top 20 amino acid substitutions), indicating that the amino acid substitutions contribute to the prediction. Moreover, including more amino acid substitutions (top 5 up to top 20) increases the contribution as expected (Supplementary figure 17). We refrain from reporting p values since highly significant p values are simply achieved with enough bootstraps.

**Supplementary tables**

**Supplementary Table 1 Covariables used in multivariable models for adjustment**

| **Adjusted factors** | **Definition** | **Reference** |
| --- | --- | --- |
| Self-reported sex | male or female | Male |
| Ethnicity | white, black, Hispano-American,  Other | White |
| Age | Age at first SRNCs (in decades) | - |
| Mode of HIV acquisition | MSM, Het., Other | MSM |
| Education (proxy for socio-economic status) | No completed school, mandatory school, higher education, other | No completed school |
| Depression | Years of self-reported depression | - |
| Use of anti-depressants | Years of use | - |
| Recreational drugs | Ever use of cocaine, heroin, or cannabis | Never |
| Use of efavirenz | Years of use | - |
| Hepatitis B | positive Hepatitis B core-antigen-antibody test and positive Hepatitis B surface-antigen-antibody test | Negative |
| Hepatitis C | positive Hepatitis C antigen-antibody test | Negative |
| Neurological disease | any neurological infections or neurological injuries (including efavirenz caused) | No disease |
| HIV-1 plasma RNA | Area under the curve over time of reported SRNCs divided by timeframe (copies/ml log10 transformed) | - |
| CD4 T cell count | Area under the curve over time of SRNCs divided by timeframe (cells/ml square-root transformed) | - |

**Supplementary Table 2 NGS sequence origin stratified by HIV subtype**

| **HIV Subtype** | **Overall** | **A** | **AE** | **AG** | **B** | **C** | **D** | **F** | **G** | **Other** |
| --- | --- | --- | --- | --- | --- | --- | --- | --- | --- | --- |
| n | 3287^a^ | 144 | 176 | 126 | 2458 | 124 | 22 | 8 | 50 | 179 |
| Proviral source,  n (%) | 1507  (45.8) | 69  (47.9) | 80 (45.5) | 68 (54.0) | 1047 (42.6) | 79 (63.7) | 8 (36.4) | 4  (50.0) | 35 (70.0) | 117 (65.4) |

^a^Number of unique samples used for NGS, from 2643 unique SHCS participants. 2232 had one, 252 had two, 100 had three, 45 had four, 13 had five, and 1 had six different NGS sequences used.

**Supplementary Table 3 NGS and partial *pol* sequence availability**

|  | **Overall** | **HIV subtype B** | **Subtype non-B** | **P** |
| --- | --- | --- | --- | --- |
| n | 8547 | 5815 | 2732 |  |
| Partial *pol* sequence, n (%) | 6966 (81.5) | 4936 (84.9) | 2030 (74.3) | <0.001 |
| Overall NGS sequence availability, n (%) | 2613 (30.6) | 1931 (33.2) | 682 (25.0) | <0.001 |
| **Co-receptor tropism^a,b^, n (%)** | | | | <0.001 |
| CXCR4 | 316 (3.7) | 221 (3.8) | 95 (3.5) |  |
| CCR5 | 1907 (22.3) | 1441 (24.8) | 466 (17.1) |  |
| Not phenotyped | 6324 (74.0) | 4153 (71.4) | 2171 (79.5) |  |
| **Gene NGS sequence availability, n (%)** | | | | |
| Env sequence^b^ | 2129 (24.9) | 1648 (28.3) | 481 (17.6) | <0.001 |
| Gag sequence | 2155 (25.2) | 1645 (28.3) | 510 (18.7) | <0.001 |
| Pol sequence | 2171 (25.4) | 1630 (28.0) | 541 (19.8) | <0.001 |
| Tat sequence | 2279 (26.7) | 1734 (29.8) | 545 (19.9) | <0.001 |
| Nef sequence | 2334 (27.3) | 1750 (30.1) | 584 (21.4) | <0.001 |
| Vpr sequence | 2304 (27.0) | 1717 (29.5) | 587 (21.5) | <0.001 |
| Vpu sequence | 2209 (25.8) | 1752 (30.1) | 457 (16.7) | <0.001 |
| Vif sequence | 2277 (26.6) | 1708 (29.4) | 569 (20.8) | <0.001 |
| Rev sequence | 2314 (27.1) | 1728 (29.7) | 586 (21.4) | <0.001 |

^a^gene2pheno was run with whole genome. ^b^total number of predicted co-receptor tropism is higher than Env sequences due to filter criteria.

**Supplementary Table 4 Subtype distribution**

| **HIV-1 Subtype^a^** | **n (%)** |
| --- | --- |
| Overall | 8547 (100) |
| A | 509 (6.0) |
| AE | 372 (4.4) |
| AG | 417 (4.9) |
| B | 5815 (68.0) |
| C | 391 (4.6) |
| D | 74 (0.9) |
| F | 75 (0.9) |
| G | 150 (1.8) |
| Other | 744 (8.7) |

^a^Subtype was determined from NGS near whole genome with Comet ^1^, partial pol with Rega ^2^, or from geno2pheno NGS V3^3^

**Supplementary Table 5 Correlation between AUC phenotypes**

|  | **Combination** | **Frequent memory loss** | **Concentration**  **difficulties** | **Cognitive slowing** |
| --- | --- | --- | --- | --- |
| **Combination** | 1 |  |  |  |
| **Frequent memory loss** | 0.93 | 1 |  |  |
| **Concentration difficulties** | 0.95 | 0.82 | 1 |  |
| **Cognitive slowing** | 0.91 | 0.75 | 0.94 | 1 |

**Supplementary Table 6 Top 50 GWAS associations among all HIV-1 subtypes**

| **Phenotype** | **Protein** | **Position** | **wt** | **mut** | **n wt** | **n mut** | **effect** | **se** | **P** |
| --- | --- | --- | --- | --- | --- | --- | --- | --- | --- |
| Frequent memory loss | Env | 641 | L | E | 1012 | 96 | 0.34 | 0.07 | 4.3*10-6 |
| Cognitive slowing | Tat | 64 | T | N | 1307 | 212 | -0.36 | 0.09 | 2.9*10-5 |
| Combination | Env | 641 | L | E | 1012 | 96 | 0.67 | 0.16 | 3.9*10-5 |
| Concentration difficulties | Tat | 64 | T | N | 1307 | 212 | -0.28 | 0.07 | 1.2*10-4 |
| Combination | Tat | 64 | T | N | 1307 | 212 | -0.47 | 0.13 | 2.5*10-4 |
| Concentration difficulties | Pol | 646 | L | P | 1244 | 82 | 0.34 | 0.09 | 2.5*10-4 |
| Concentration difficulties | Gag | 372 | N | G | 1309 | 130 | -0.31 | 0.09 | 2.8*10-4 |
| Concentration difficulties | Rev | 18 | L | I | 1307 | 547 | -0.26 | 0.07 | 2.8*10-4 |
| Concentration difficulties | Env | 134 | L | W | 403 | 113 | 0.30 | 0.09 | 3.8*10-4 |
| Cognitive slowing | Gag | 232 | R | K | 2078 | 65 | 0.43 | 0.12 | 4.3*10-4 |
| Frequent memory loss | Tat | 64 | T | N | 1307 | 212 | -0.21 | 0.06 | 4.5*10-4 |
| Cognitive slowing | Pol | 9 | P | Q | 1550 | 372 | 0.32 | 0.09 | 7.1*10-4 |
| Cognitive slowing | Env | 19 | I | T | 699 | 675 | -0.18 | 0.05 | 9.0*10-4 |
| Concentration difficulties | Gag | 373 | S | A | 1055 | 101 | -0.32 | 0.10 | 1.0*10-3 |
| Combination | Pol | 646 | L | P | 1244 | 82 | 0.59 | 0.18 | 1.3*10-3 |
| Cognitive slowing | Gag | 252 | N | G | 981 | 117 | 0.30 | 0.09 | 1.4*10-3 |
| Concentration difficulties | Env | 275 | E | A | 1341 | 211 | -0.22 | 0.07 | 1.4*10-3 |
| Cognitive slowing | Vpr | 37 | P | L | 991 | 83 | 0.32 | 0.10 | 1.4*10-3 |
| Concentration difficulties | Gag | 387 | R | K | 1698 | 382 | 0.19 | 0.06 | 1.4*10-3 |
| Combination | Rev | 18 | L | I | 1307 | 547 | -0.41 | 0.13 | 1.5*10-3 |
| Frequent memory loss | Rev | 14 | K | T | 1151 | 77 | -0.30 | 0.09 | 1.6*10-3 |
| Concentration difficulties | Rev | 91 | T | N | 2048 | 103 | 0.26 | 0.08 | 1.6*10-3 |
| Cognitive slowing | Rev | 18 | L | I | 1307 | 547 | -0.26 | 0.08 | 1.6*10-3 |
| Cognitive slowing | Vif | 181 | K | R | 1717 | 474 | -0.25 | 0.08 | 1.9*10-3 |
| Frequent memory loss | Rev | 18 | L | I | 1307 | 547 | -0.18 | 0.06 | 2.0*10-3 |
| Cognitive slowing | Gag | 441 | H | N | 1574 | 226 | 0.29 | 0.09 | 2.1*10-3 |
| Frequent memory loss | Pol | 332 | D | E | 1257 | 825 | 0.12 | 0.04 | 2.4*10-3 |
| Combination | Rev | 91 | T | N | 2048 | 103 | 0.47 | 0.16 | 2.6*10-3 |
| Combination | Gag | 387 | R | K | 1698 | 382 | 0.34 | 0.11 | 2.6*10-3 |
| Combination | Env | 149 | M | S | 463 | 119 | -0.52 | 0.17 | 2.7*10-3 |
| Cognitive slowing | Rev | 91 | T | N | 2048 | 103 | 0.27 | 0.09 | 2.7*10-3 |
| Combination | Gag | 232 | R | K | 2078 | 65 | 0.64 | 0.21 | 2.8*10-3 |
| Concentration difficulties | Pol | 590 | V | I | 1270 | 311 | -0.17 | 0.06 | 2.8*10-3 |
| Frequent memory loss | Gag | 387 | R | K | 1698 | 382 | 0.16 | 0.05 | 2.8*10-3 |
| Cognitive slowing | Pol | 646 | L | P | 1244 | 82 | 0.31 | 0.10 | 2.9*10-3 |
| Frequent memory loss | Env | 187 | D | K | 654 | 68 | 0.27 | 0.09 | 2.9*10-3 |
| Cognitive slowing | Nef | 8 | S | N | 817 | 70 | 0.32 | 0.11 | 3.1*10-3 |
| Combination | Pol | 332 | D | E | 1257 | 825 | 0.26 | 0.09 | 3.2*10-3 |
| Combination | Env | 683 | K | R | 1672 | 375 | -0.28 | 0.09 | 3.2*10-3 |
| Combination | Rev | 14 | K | T | 1151 | 77 | -0.60 | 0.20 | 3.3*10-3 |
| Frequent memory loss | Env | 190 | S | N | 1113 | 150 | -0.19 | 0.07 | 3.3*10-3 |
| Cognitive slowing | Gag | 441 | H | S | 1574 | 179 | 0.27 | 0.09 | 3.4*10-3 |
| Cognitive slowing | Env | 134 | L | W | 403 | 113 | 0.28 | 0.10 | 3.5*10-3 |
| Frequent memory loss | Env | 46 | K | R | 1462 | 238 | 0.17 | 0.06 | 3.5*10-3 |
| Concentration difficulties | Vif | 134 | E | N | 1848 | 64 | -0.40 | 0.14 | 3.5*10-3 |
| Cognitive slowing | Gag | 387 | R | K | 1698 | 382 | 0.19 | 0.07 | 3.7*10-3 |
| Concentration difficulties | Vif | 155 | T | A | 1386 | 150 | 0.20 | 0.07 | 3.8*10-3 |
| Cognitive slowing | Vif | 63 | K | E | 1184 | 81 | -0.39 | 0.13 | 3.9*10-3 |
| Combination | Gag | 373 | S | A | 1055 | 101 | -0.50 | 0.17 | 4.0*10-3 |
| Concentration difficulties | Env | 464 | T | N | 630 | 300 | -0.17 | 0.06 | 4.1*10-3 |

**Supplementary Table 7 Top 50 GWAS associations restricted to HIV-1 subtype B**

| **Phenotype** | **Protein** | **Position** | **wt** | **mut** | **n wt** | **n mut** | **effect** | **se** | **P** |
| --- | --- | --- | --- | --- | --- | --- | --- | --- | --- |
| Concentration difficulties | Rev | 18 | L | I | 1211 | 154 | -0.36 | 0.08 | 1.7*10-5 |
| Concentration difficulties | Tat | 64 | T | N | 1238 | 157 | -0.34 | 0.08 | 3.3*10-5 |
| Concentration difficulties | Env | 464 | T | N | 432 | 209 | -0.29 | 0.07 | 8.5*10-5 |
| Cognitive slowing | Tat | 64 | T | N | 1238 | 157 | -0.37 | 0.10 | 1.8*10-4 |
| Combination | Tat | 64 | T | N | 1238 | 157 | -0.54 | 0.15 | 2.0*10-4 |
| Frequent memory loss | Env | 641 | L | E | 884 | 75 | 0.31 | 0.08 | 2.6*10-4 |
| Frequent memory loss | Tat | 64 | T | N | 1238 | 157 | -0.24 | 0.07 | 4.1*10-4 |
| Frequent memory loss | Pol | 332 | D | E | 1121 | 315 | 0.16 | 0.05 | 6.9*10-4 |
| Concentration difficulties | Env | 275 | E | A | 949 | 181 | -0.24 | 0.07 | 1.1*10-3 |
| Combination | Pol | 332 | D | E | 1121 | 315 | 0.33 | 0.10 | 1.1*10-3 |
| Frequent memory loss | Env | 534 | S | A | 1450 | 63 | 0.29 | 0.09 | 1.2*10-3 |
| Combination | Env | 464 | T | N | 432 | 209 | -0.43 | 0.14 | 1.3*10-3 |
| Combination | Env | 724 | P | Q | 870 | 350 | -0.34 | 0.11 | 1.3*10-3 |
| Combination | Rev | 18 | L | I | 1211 | 154 | -0.47 | 0.15 | 1.6*10-3 |
| Cognitive slowing | Rev | 18 | L | I | 1211 | 154 | -0.30 | 0.10 | 1.9*10-3 |
| Frequent memory loss | Env | 700 | T | S | 754 | 79 | 0.26 | 0.08 | 1.9*10-3 |
| Cognitive slowing | Vif | 98 | V | I | 1528 | 111 | -0.37 | 0.12 | 1.9*10-3 |
| Concentration difficulties | Gag | 373 | S | A | 946 | 81 | -0.34 | 0.11 | 2.1*10-3 |
| Combination | Env | 641 | L | E | 884 | 75 | 0.58 | 0.19 | 2.2*10-3 |
| Frequent memory loss | Gag | 46 | V | L | 1317 | 127 | -0.22 | 0.07 | 2.2*10-3 |
| Cognitive slowing | Nef | 203 | Y | F | 1351 | 192 | 0.22 | 0.07 | 2.4*10-3 |
| Frequent memory loss | Env | 464 | T | N | 432 | 209 | -0.19 | 0.06 | 2.8*10-3 |
| Frequent memory loss | Env | 47 | E | D | 1195 | 133 | -0.21 | 0.07 | 2.8*10-3 |
| Frequent memory loss | Env | 190 | S | N | 982 | 132 | -0.21 | 0.07 | 2.9*10-3 |
| Concentration difficulties | Env | 724 | P | Q | 870 | 350 | -0.17 | 0.06 | 2.9*10-3 |
| Concentration difficulties | Gag | 372 | N | G | 1111 | 104 | -0.29 | 0.10 | 3.0*10-3 |
| Frequent memory loss | Env | 817 | A | V | 1113 | 75 | -0.27 | 0.09 | 3.3*10-3 |
| Frequent memory loss | Rev | 18 | L | I | 1211 | 154 | -0.20 | 0.07 | 3.5*10-3 |
| Cognitive slowing | Env | 724 | P | Q | 870 | 350 | -0.18 | 0.06 | 3.6*10-3 |
| Concentration difficulties | Env | 134 | L | W | 351 | 101 | 0.26 | 0.09 | 4.0*10-3 |
| Cognitive slowing | Env | 19 | I | T | 633 | 474 | -0.17 | 0.06 | 4.2*10-3 |
| Concentration difficulties | Gag | 441 | H | Y | 1321 | 64 | -0.38 | 0.13 | 4.5*10-3 |
| Combination | Env | 700 | T | S | 754 | 79 | 0.51 | 0.18 | 5.3*10-3 |
| Frequent memory loss | Env | 496 | V | I | 890 | 504 | -0.12 | 0.04 | 5.4*10-3 |
| Frequent memory loss | Env | 396 | N | P | 581 | 74 | -0.26 | 0.10 | 5.6*10-3 |
| Frequent memory loss | Env | 344 | Q | K | 938 | 252 | -0.15 | 0.05 | 5.8*10-3 |
| Combination | Pol | 661 | I | L | 1362 | 106 | -0.47 | 0.17 | 5.8*10-3 |
| Frequent memory loss | Env | 796 | W | C | 1101 | 103 | -0.22 | 0.08 | 5.8*10-3 |
| Concentration difficulties | Env | 818 | T | I | 1101 | 295 | 0.15 | 0.05 | 6.2*10-3 |
| Concentration difficulties | Pol | 828 | I | V | 1270 | 134 | 0.20 | 0.07 | 6.5*10-3 |
| Frequent memory loss | Vif | 29 | M | I | 1233 | 286 | 0.13 | 0.05 | 6.6*10-3 |
| Concentration difficulties | Env | 700 | T | S | 754 | 79 | 0.26 | 0.10 | 6.7*10-3 |
| Concentration difficulties | Pol | 661 | I | L | 1362 | 106 | -0.25 | 0.09 | 6.7*10-3 |
| Concentration difficulties | Env | 463 | N | S | 810 | 243 | 0.17 | 0.06 | 6.8*10-3 |
| Combination | Nef | 188 | R | K | 1041 | 135 | 0.39 | 0.15 | 6.8*10-3 |
| Combination | Vif | 29 | M | I | 1233 | 286 | 0.28 | 0.10 | 6.9*10-3 |
| Combination | Env | 411 | N | E | 847 | 115 | -0.45 | 0.17 | 7.4*10-3 |
| Frequent memory loss | Env | 724 | P | Q | 870 | 350 | -0.13 | 0.05 | 7.8*10-3 |
| Cognitive slowing | Env | 138 | T | S | 526 | 133 | 0.24 | 0.09 | 8.0*10-3 |
| Combination | Vpr | 77 | R | H | 749 | 154 | 0.44 | 0.16 | 8.0*10-3 |

**Supplementary Table 8 Explained genetic variance of the first ten principal components**

| Gene | Sequence type | Explained variance first 10 PCs | |
| --- | --- | --- | --- |
|  |  | Subtype B | Subtype all |
| *env* | Near whole genome | 3.65% | 5.7% |
| *gag* | Near whole genome | 4.41% | 7.37% |
| *pol* | Near whole genome | 3.81% | 8.32% |
| *pol* | Partial *pol* | 3.48% | 6.17% |
| *nef* | Near whole genome | 6.17% | 7.65% |
| *vif* | Near whole genome | 6.93% | 8.42% |
| *vpr* | Near whole genome | 10.23% | 10.88% |
| *vpu* | Near whole genome | 9.3% | 11.02% |
| *tat* | Near whole genome | 9.14% | 10.56% |
| *rev* | Near whole genome | 8.67% | 10.55% |

**Supplementary Table 9 Effect of minor nucleotide frequency on SRNCs**

| **Amino acid** | **Nucleotide codon  pos. 1** | **Nucleotide**  **codon**  **pos. 2** | **Nucleotide**  **codon**  **pos. 3** | **Phenotype** | **Latent marginal effect of nucleotide frequency^a^** |
| --- | --- | --- | --- | --- | --- |
| **All subtypes** | | | | | |
| Env gp160 L641E | C/T to G | T to A | - | Frequent memory loss | 1.11 (95% CI -7.9, 10.12) |
| (interaction: G * A) |  |  |  |  |  |
| Tat T64N | - | C to A | - | Cognitive slowing | -0.27 (95% CI -1.01, 0.47) |
| **Subtype B** | | | | | |
| Rev L18I | C to A | - | - | Concentration difficulties | -0.64 (95% CI -2.35, 1.07). |
| Tat T64N | - | C to A | - | Concentration difficulties | -0.27 (95% CI -1.81, 1.27) |
| Env gp160 T464N | - | C to A | - | Concentration difficulties | -0.75 (95% CI -2.08, 0.57) |
| Env gp160 L641E | C/T to G | T to A | - | Frequent memory loss | 1.11 (95% CI -7.9, 10.12) |
| (interaction: G * A) |  |  |  |  |  |
| Tat T64N | - | C to A | - | Cognitive slowing | -0.27 (95% CI -1.01, 0.47) |

^a^(Analyses restricted to those withmajor amino acid)

**Supplementary Table 10 Associations of Env mutations found by previous studies**

| **Env Mutation** | **Original study** | **Associated measure** | **Effect direction** | **Present study mutation** | **Present study**  ***P* value** | **Phenotype associated** | **Original study** |
| --- | --- | --- | --- | --- | --- | --- | --- |
| R315K | Holman & Gabuzda ^19^ | HAND | + | R315K | p=0.04 | Concentration difficulties | - |
| *291S | Ogishi & Yotsuyanagi ^20^ | HAND | - | A291S | p<0.005 | Concentration difficulties, cognitive slowing, frequent memory loss, combination | - |
| *340N | Ogishi & Yotsuyanagi ^20^ | HAND | - | D340N | p<0.05 | Frequent memory loss | + |
| E293K | Strain ^21^ | CSF | + | E293N  (not 293K) | p<0.05 | Concentration difficulties, cognitive slowing, frequent memory loss, combination | + |
| *308H | Strain ^21^ | CSF | + | R308H | p<0.05 | Concentration difficulties, cognitive slowing, frequent memory loss, combination | - |

**Supplementary Figures**

**Supplementary Figure 1** Related to figure 3 but restricted to people with HIV (PWH) with HIV-1 subtype B. Broad sense heritability of the self-reported neurocognitive complaints (SRNC) phenotypes cognitive slowing, concentration difficulties, frequent memory loss, and their combination approximated by intraclass correlation (ICC). ICC is estimated by comparison of a mixed tobit model using phylogenetic cluster under specified thresholds as random effects and a tobit model without mixed effects. The number above the bars indicate the test statistic (*test statistic: chi-bar-squared) and p value of the respective ICC. Phenotypes are calculated as the area under the curve (AUC) of longitudinally measured cognitive slowing, concentration difficulties, frequent memory loss, or the combination of all three. Analysis was done on 4,936 partial *pol* (subtype B) sequences. Absence of confidence intervals are non-converged models.

****Supplementary Figure 2** Related to figure 4 but restricted to people with HIV (PWH) with HIV-1 subtype B. Genome wide association study of the HIV-1 genome and associations with self-reported neurocognitive complaints (SRNC) in PWH. SRNCs are defined as the area under the curve (AUC) of longitudinally measured cognitive slowing, concentration difficulties, frequent memory loss, or the combination of all three. The p values were calculated with a multivariable tobit model (test statistic: z).

**Supplementary Figure 3** Quantile-quantile plot of negative log10 *P* values of genome wide association study (GWAS) analysis including all HIV-1 subtypes (Figure 4). Figure was created with the R package qqman.^22^ Genomic inflation factor lambda: 0.98.

**Supplementary Figure 4** Quantile-quantile plot of negative log10 *P* values of genome wide association study (GWAS) analysis restricted to HIV-1 subtype B (Supplementary Figure 2). Figure was created with the R package qqman.^22^ Genomic inflation factor lambda: 1.01.

**Supplementary Figure 5** Null model for genome wide association study (GWAS) adjustment for self-reported neurocognitive complaints (SRNC) phenotype “combination” including all HIV-1 subtypes. Shown are the latent marginal effects of a tobit model with a lower censoring of 0.

**Supplementary Figure 6** Null model for genome wide association study (GWAS) adjustment for self-reported neurocognitive complaints (SRNC) phenotype “frequent memory loss” including all HIV-1 subtypes. Shown are the latent marginal effects of a tobit model with a lower censoring of 0.

**Supplementary Figure 7** Null model for genome wide association study (GWAS) adjustment for self-reported neurocognitive complaints (SRNC) phenotype “concentration difficulties” including all HIV-1 subtypes. Shown are the latent marginal effects of a tobit model with a lower censoring of 0.

**Supplementary Figure 8** Null model for genome wide association study (GWAS) adjustment for self-reported neurocognitive complaints (SRNC) phenotype “cognitive slowing” including all HIV-1 subtypes. Shown are the latent marginal effects of a tobit model with a lower censoring of 0.

**Supplementary Figure 9** Null model for genome wide association study (GWAS) adjustment for self-reported neurocognitive complaints (SRNC) phenotype “combination” restricted to HIV-1 subtype B. Shown are the latent marginal effects of a tobit model with a lower censoring of 0.

**Supplementary Figure 10** Null model for genome wide association study (GWAS) adjustment for self-reported neurocognitive complaints (SRNC) phenotype “frequent memory loss” restricted to HIV-1 subtype B. Shown are the latent marginal effects of a tobit model with a lower censoring of 0.

**Supplementary Figure 11** Null model for genome wide association study (GWAS) adjustment for self-reported neurocognitive complaints (SRNC) phenotype “concentration difficulties” restricted to HIV-1 subtype B. Shown are the latent marginal effects of a tobit model with a lower censoring of 0.

**Supplementary Figure 12** Null model for genome wide association study (GWAS) adjustment for self-reported neurocognitive complaints (SRNC) phenotype “cognitive slowing” including all HIV-1 subtypes. Shown are the latent marginal effects of a tobit model with a lower censoring of 0.

**
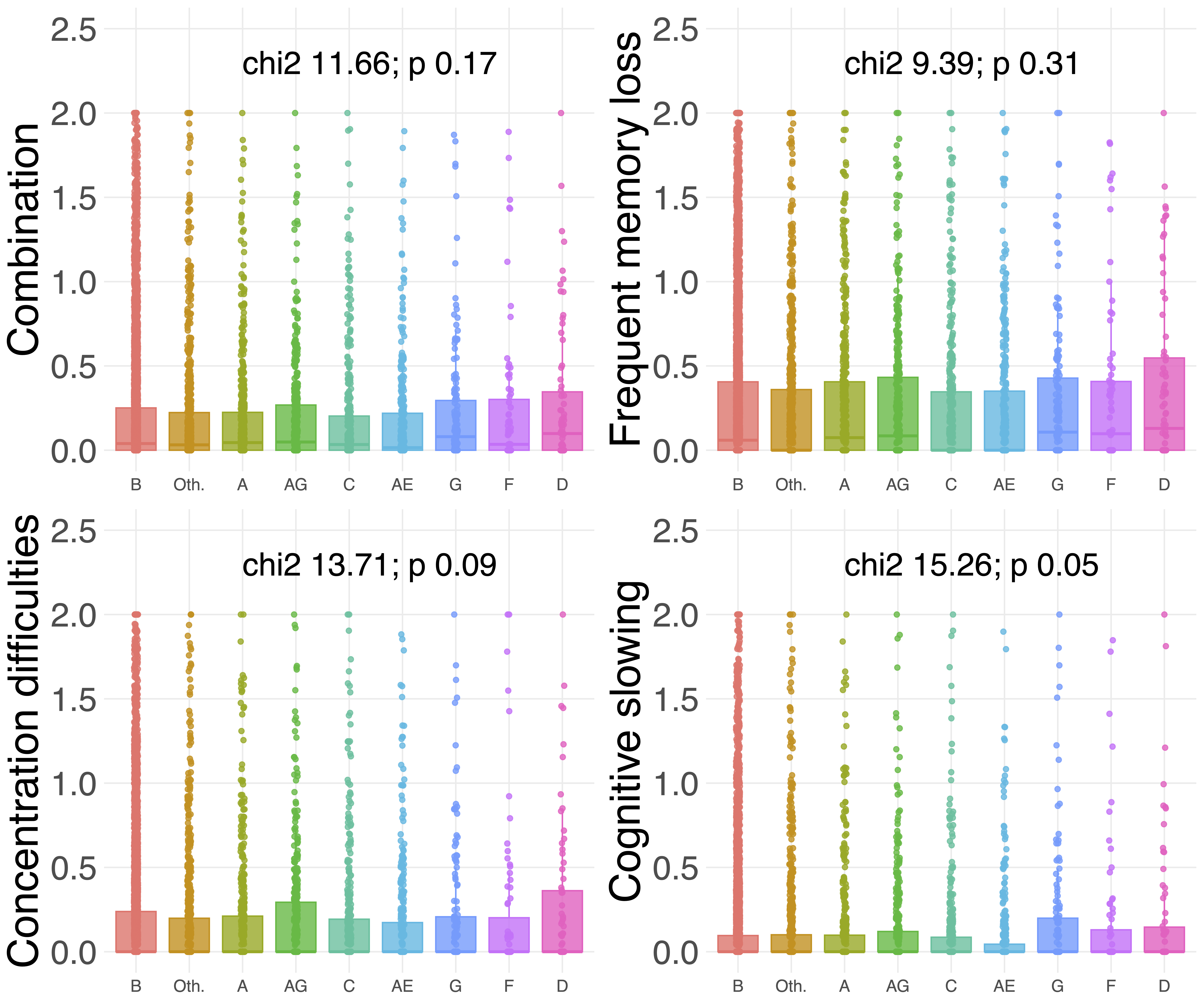
**

**Supplementary Figure 13** Self-reported neurocognitive complaints (SRNCs) in association with HIV-1 subtype among people with HIV (PWH) from all ethnicities. SRNCs are defined as the area under the curve (AUC) of longitudinally measured cognitive slowing, concentration difficulties, frequent memory loss, or the combination of all three, over the follow up period. Subtype is predicted on partial *pol* sequences with Rega.^2^ The shown p values are estimated with a likelihood ratio test (test statistic: chi2) between a null model and a univariable tobit model (with subtype B vs. different non-B as predictor).


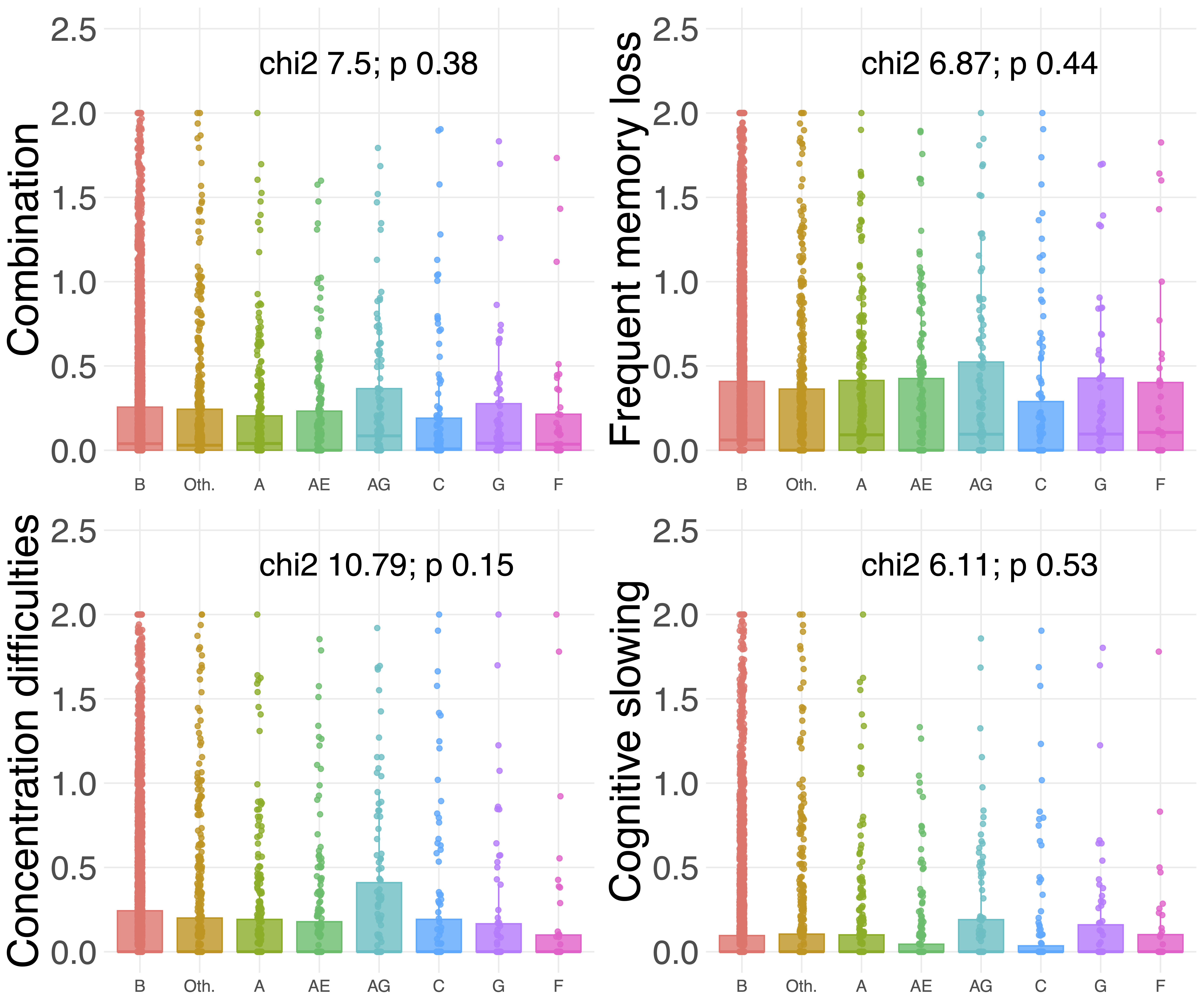


**Supplementary Figure 14** Self-reported neurocognitive complaints (SRNCs) in association with HIV-1 subtype among people with HIV (PWH) of white ethnicity. SRNCs are defined as the area under the curve (AUC) of longitudinally measured cognitive slowing, concentration difficulties, frequent memory loss, or the combination of all three, over the follow up period. Subtype is predicted on partial *pol* sequences with Rega.^2^ The shown p values are estimated with a likelihood ratio test (test statistic: chi2) between a null model and a univariable tobit model (with subtype B vs. different non-B as predictor).

**Supplementary Figure 15** Self-reported neurocognitive complaints (SRNCs) in association with HIV-1 coreceptor tropism among all HIV-1 subtypes. SRNCs are defined as the area under the curve (AUC) of longitudinally measured cognitive slowing, concentration difficulties, frequent memory loss, or the combination of all three, over the follow up period. Coreceptor tropism is predicted with geno2pheno, classification cutoff is defined as >5.75% for CCR5 only and <=5.75% for CXCR4 capable. ^3^ P values and effect size are estimated with a univariable tobit model (test statistic: z).

**Supplementary Figure 16** Self-reported neurocognitive complaints (SRNCs) in association with HIV-1 coreceptor tropism restricted to HIV-1 subtype B. SRNCs are defined as the area under the curve (AUC) of longitudinally measured cognitive slowing, concentration difficulties, frequent memory loss, or the combination of all three, over the follow up period. Coreceptor tropism is predicted with geno2pheno, classification cutoff is defined as >5.75% for CCR5 only and <=5.75% for CXCR4 capable. ^3^ P values and effect size are estimated with a univariable tobit model (test statistic: z).

**Supplementary Figure 17** Distribution density of R^2^ difference between the full model (including top associated amino acid substitutions from a genome wide associations study (GWAS)) and partial model (including only covariables) with self-reported neurocognitive complaints (SRNC) as the outcome from 4000 bootstraps of 10fold cross validation with the R package “tobitnet”. Vertical lines indicate the median.

**Supplementary Figure 18** related to figure 4, including all HIV-1 subtypes. Unadjusted (i.e., only with first ten principal components for population structure) genome wide association study (GWAS) of the HIV-1 genome (including all subtypes) and associations with self-reported neurocognitive complaints (SRNC) in people with HIV (PWH). SRNCs are defined as the area under the curve (AUC) of longitudinally measured cognitive slowing, concentration difficulties, frequent memory loss, or the combination of all three. The *P* values were calculated with a multivariable tobit model. The p values were calculated with a multivariable tobit model (test statistic: z).

**Supplementary Figure 19** related to supplementary figure 2, i.e., restricted to people with HIV (PWH) with HIV-1 subtype B. Unadjusted (i.e., only with first ten principal components for population structure) genome wide association study (GWAS) of the HIV-1 genome and associations with self-reported neurocognitive complaints (SRNC) in PWH. SRNCs are defined as the area under the curve (AUC) of longitudinally measured cognitive slowing, concentration difficulties, frequent memory loss, or the combination of all three. The p values were calculated with a multivariable tobit model (test statistic: z).

**Supplementary References**

1. Struck D, Lawyer G, Ternes AM, Schmit JC, Bercoff DP. COMET: adaptive context-based modeling for ultrafast HIV-1 subtype identification. *Nucleic Acids Res*. 2014;42(18):e144-e144. doi:10.1093/nar/gku739

2. Pineda-Peña AC, Faria NR, Imbrechts S, et al. Automated subtyping of HIV-1 genetic sequences for clinical and surveillance purposes: Performance evaluation of the new REGA version 3 and seven other tools. *Infection, Genetics and Evolution*. 2013;19:337-348. doi:10.1016/j.meegid.2013.04.032

3. Lengauer T, Sander O, Sierra S, Thielen A, Kaiser R. Bioinformatics prediction of HIV coreceptor usage. *Nat Biotechnol*. 2007;25(12):1407-1410. doi:10.1038/nbt1371

4. Gulick RM, Lalezari J, Goodrich J, et al. Maraviroc for Previously Treated Patients with R5 HIV-1 Infection. *New England Journal of Medicine*. 2008;359(14):1429-1441. doi:10.1056/NEJMoa0803152

5. Kleiber C, Zeileis A. *Applied Econometrics with {R}*. Springer-Verlag; 2008. https://cran.r-project.org/package=AER

6. Carlisle LA, Turk T, Kusejko K, et al. Viral Diversity Based on Next-Generation Sequencing of HIV-1 Provides Precise  Estimates of Infection Recency and Time Since Infection. *J Infect Dis*. 2019;220(2):254-265. doi:10.1093/infdis/jiz094

7. Baeten JM, Chohan B, Lavreys L, et al. HIV‐1 Subtype D Infection Is Associated with Faster Disease Progression than Subtype A in Spite of Similar Plasma HIV‐1 Loads. *J Infect Dis*. 2007;195(8):1177-1180. doi:10.1086/512682

8. Sacktor N, Nakasujja N, Skolasky RL, et al. HIV Subtype D Is Associated with Dementia, Compared with Subtype A, in Immunosuppressed Individuals at Risk of Cognitive Impairment in Kampala, Uganda. *Clinical Infectious Diseases*. 2009;49(5):780-786. doi:10.1086/605284

9. Boivin MJ, Ruel TD, Boal HE, et al. HIV-subtype A is associated with poorer neuropsychological performance compared with subtype D in antiretroviral therapy-naive Ugandan children. *AIDS*. 2010;24(8):1163-1170. doi:10.1097/QAD.0b013e3283389dcc

10. Jumare J, Ndembi N, El-Kamary SS, et al. Cognitive Function Among Antiretroviral Treatment–Naive Individuals Infected With Human Immunodeficiency Virus Type 1 Subtype G Versus CRF02_AG in Nigeria. *Clinical Infectious Diseases*. 2018;66(9):1448-1453. doi:10.1093/cid/cix1019

11. He J, Chen Y, Farzan M, et al. CCR3 and CCR5 are co-receptors for HIV-1 infection of microglia. *Nature*. 1997;385(6617):645-649. doi:10.1038/385645a0

12. Albright A V., Shieh JTC, Itoh T, et al. Microglia Express CCR5, CXCR4, and CCR3, but of These, CCR5 Is the Principal Coreceptor for Human Immunodeficiency Virus Type 1 Dementia Isolates. *J Virol*. 1999;73(1):205-213. doi:10.1128/JVI.73.1.205-213.1999

13. Gray L, Roche M, Churchill MJ, et al. Tissue-Specific Sequence Alterations in the Human Immunodeficiency Virus Type 1 Envelope Favoring CCR5 Usage Contribute to Persistence of Dual-Tropic Virus in the Brain. *J Virol*. 2009;83(11):5430-5441. doi:10.1128/JVI.02648-08

14. Riviere-Cazaux C, Cornell J, Shen Y, Zhou M. The role of CCR5 in HIV-associated neurocognitive disorders. *Heliyon*. 2022;8(7):e09950. doi:10.1016/j.heliyon.2022.e09950

15. Smith LK, Babcock IW, Minamide LS, Shaw AE, Bamburg JR, Kuhn TB. Direct interaction of HIV gp120 with neuronal CXCR4 and CCR5 receptors induces cofilin-actin rod pathology via a cellular prion protein- and NOX-dependent mechanism. *PLoS One*. 2021;16(3):e0248309. doi:10.1371/journal.pone.0248309

16. Morris SR, Woods SP, Deutsch R, et al. Dual-mixed HIV-1 coreceptor tropism and HIV-associated neurocognitive deficits. *J Neurovirol*. 2013;19(5):488-494. doi:10.1007/s13365-013-0203-9

17. Bleul CC, Wu L, Hoxie JA, Springer TA, Mackay CR. The HIV coreceptors CXCR4 and CCR5 are differentially expressed and regulated on human T lymphocytes. *Proceedings of the National Academy of Sciences*. 1997;94(5):1925-1930. doi:10.1073/pnas.94.5.1925

18. Jacobson T, Zou H. High-Dimensional Censored Regression via the Penalized Tobit Likelihood. *Journal of Business & Economic Statistics*. Published online March 15, 2023:1-12. doi:10.1080/07350015.2023.2182309

19. Holman A, Gabuzda D. A Machine Learning Approach for Identifying Amino Acid Signatures in the HIV Env Gene Predictive of Dementia. *PLoS One*. 2012;7:e49538. doi:10.1371/journal.pone.0049538

20. Ogishi M, Yotsuyanagi H. Prediction of HIV-associated neurocognitive disorder (HAND) from three genetic features of envelope gp120 glycoprotein. *Retrovirology*. 2018;15(1):12. doi:10.1186/s12977-018-0401-x

21. Strain MC, Letendre S, Pillai SK, et al. Genetic composition of human immunodeficiency virus type 1 in cerebrospinal fluid  and blood without treatment and during failing antiretroviral therapy. *J Virol*. 2005;79(3):1772-1788. doi:10.1128/JVI.79.3.1772-1788.2005

22. D. Turner S. qqman: an R package for visualizing GWAS results using Q-Q and manhattan plots. *J Open Source Softw*. 2018;3(25):731. doi:10.21105/joss.00731
